# Locations of Physical Activity: Where Are Children, Adolescents, and Adults Physically Active? A Systematic Review

**DOI:** 10.3390/ijerph18031240

**Published:** 2021-01-30

**Authors:** Anne Kelso, Anne K Reimers, Karim Abu-Omar, Kathrin Wunsch, Claudia Niessner, Hagen Wäsche, Yolanda Demetriou

**Affiliations:** 1 Department of Sport and Health Sciences, Technical University of Munich, Georg-Brauchle-Ring 62, 80992 Munich, Germany; yolanda.demetriou@tum.de; 2 Department of Sport Science and Sport, University Erlangen-Nuremberg, Gebbertstrasse 123b, 91058 Erlangen, Germany; anne.reimers@fau.de (A.K.R.); karim.abu-omar@fau.de (K.A.-O.); 3 Institute of Sports and Sports Science, Karlsruhe Institute of Technology (KIT), Engler-Bunte-Ring 15, 76131 Karlsruhe, Germany; kathrin.wunsch@kit.edu (K.W.); claudia.niessner@kit.edu (C.N.); hagen.waesche@kit.edu (H.W.)

**Keywords:** behavior setting, environment, places, exercise, sedentary time, GPS, GIS

## Abstract

The aim of this systematic review was to examine where physical activity (PA) takes place and how much time children, adolescents and adults spend being physically active within the identified locations. A systematic literature search was carried out in five electronic databases (PubMed, CINAHL, SPORTDiscus, PsycInfo, Scopus). For inclusion, primary studies had to identify locations of PA using device-based or self-report tools, whereas minutes of PA had to be examined using device-based tools only. Thirty-two studies were included, methodological quality and sex/gender sensitivity of the studies were assessed. The narrative data synthesis revealed that the highest average amount of daily moderate-to-vigorous PA was found in home and recreational locations, followed by school and neighborhood locations. In adults, highest average amount of daily moderate-to-vigorous PA was found in neighborhood and home locations followed by workplace and recreational locations. The majority of studies had a low risk of bias in four out of six domains; eight studies reported significant sex/gender differences in location-based PA. The results indicate that different locations are used for PA to a varying degree across the lifespan. Future research on the promotion of PA should focus on location-specific design features that encourage children, adolescents and adults to be physically active.

## 1. Introduction

Although participation in regular exercise and physical activity (PA) has been shown to improve various health outcomes, including cardiovascular health, muscular fitness, bone health, mental health and cognitive performance [1,2], 28% of adults and 81% of school-aged children and adolescents do not engage in sufficient PA [1]. Additionally, children, adolescents and adults spend prolonged time in sedentary behaviors, which have been associated with detrimental health outcomes independent of PA, including overweight and obesity, cardiovascular diseases and type 2 diabetes [3,4,5,6].

To understand PA and sedentary behavior, various elements have to be considered. From a systems perspective, individual health behavior is a function of the individual and the environment the individual lives in [7]. Hence, the personal characteristics of individuals and environmental structures in which an individual is embedded interact with each other [8]. In addition to social structures (e.g., socio-cultural norms, socio-economic status, social networks), it is physical infrastructures (e.g., cities, parks, buildings, recreational/sports facilities) that enable or hinder PA. Settings such as the neighborhood, workplaces or schools are based on site-specific physical infrastructures, which represent the locations for individual PA, and social interaction.

The social–ecological paradigm is in line with the systems perspective, emphasizing multiple levels of influence on individual health behavior [9]. According to Sallis et al. [10], determinants of PA comprise intrapersonal characteristics (e.g., demographics, biological/psychological factors) and various environmental aspects (policy, natural, socio-cultural, information, and perceived environment) that explain active living in various domains (active recreation/transport, household/occupational activities) and behavior settings (e.g., home, neighborhood, school, workplace, recreational sites). Various ecological models of health behavior have been proposed and have been shown to be useful not only to understand individual behavior but also to inform effective strategies and multilevel interventions for PA promotion [9]. By focusing on various environments and their essential role in understanding PA and sedentary behavior, ecological models underline the importance of physical infrastructure or the locations of PA.

While many studies focused on environmental features promoting or hindering PA [11,12,13], it is also necessary to know where PA occurs. In their systematic review, Tcymbal et al. [14] highlight the effects of built environment features on PA and suggest that physical infrastructure improvements, e.g., the creation or renovation of parks, may be promising tools to promote PA. However, insights into the locations that are actually used (or have the potential to be used) for PA are needed to develop more effective strategies to support active living. In this regard, locations of active living represent the physical infrastructure that is an essential part of PA behavior settings within the environment of individuals.

Given the frequently reported differences in PA levels [15,16] and the evidence of differences in PA preferences and underlying mechanisms associated with PA between males and females [17,18], research on PA locations should consider potential differences by sex (biological construct) and/or gender (cultural construct) [19]. For example, a qualitative study showed that exercise practices and mobility in gyms differ between men and women and that women tend to minimize their use of time and space in gyms [20]. Additionally, females are more likely than males to identify a lack of convenient places as barriers of PA [21].

A key challenge in gaining insights into the use of physical locations for PA lies in its measurement. To understand where and to what extent individuals are physically active, researchers need to capture different types of data—on PA and on the physical location where PA takes place. Accelerometers, for example, measure intensities and durations of PA by classifying activity counts accumulated in a given time interval using appropriate cut-points into sedentary, light, or moderate to vigorous PA, yet fail to capture contextual detail [22]. Gaining contextual information of PA has often been based on self-reported perception without information on actual use of the environment [23]. However, advances in computer software and digital data provide diverse tools to measure physical environmental characteristics, e.g., characteristics of the places that people inhabit can be found via Geographic Information Systems (GIS) and when and where PA occurs via Global Positioning Systems (GPS) [23,24]. The combination of device-based measurements of PA and GPS, GIS or self-reported contextual data provide detailed information on the space–time–activity patterns at the individual level. However, the capacity, and/or limitations of each measurement tool should be considered [25,26].

Previous reviews in this field have examined where PA occurs by focusing on primary studies using GPS and GIS data combined with device-based measurements of PA [27] and focused on a specific population (i.e., 5 to 18-year-old children and adolescents) [23]. In both of these reviews, sex/gender aspects were not considered in depth. Additionally, the domains leisure time and active transportation were considered as locations of PA [23,27]. However, these domains are geographically inconclusive. Furthermore, these reviews also included primary studies that only examined the proportion of time engaged in PA in specific locations, which provide less information than the absolute amount of minutes of PA, and did not require a minimum data collection period (i.e., wear time of measurement devices) [28].

To gain a more detailed insight into the use of specific locations for PA, the aim of this systematic review was to examine children’s, adolescents’, and adults’ time (in minutes) engaged in PA at locations that can be precisely identified within a physical environment. Specific locations, such as parks, playgrounds or schools and work buildings, enable a clear identification within a spatial context. In addition, this review covered the entire lifespan, analyzing PA in different age groups from childhood to adulthood based on device-based measurements of PA combined with self-reported and device-based measurements of physical locations. Due to the above-mentioned differences in PA between males and females and the need to incorporate sex and gender at various stages of the research process in health research [29], sex/gender aspects in primary studies were considered concerning the study concept, study design, presentation of findings, and interpretation of findings. Consistent with an ecological approach to modifying health behaviors, identifying key locations of PA in males and females can help future intervention developers, city planners and governments focus their strategies on relevant locations to increase habitual PA in individuals of all ages.

## 2. Materials and Methods 

This systematic review followed the Preferred Reporting Items for Systematic Reviews and Meta-Analyses (PRISMA) statement (see Table S1) [30] and was registered to the international prospective register of systematic reviews PROSPERO [31] on 28 April 2020 (registration number: CRD42020150201).

Due to the different terminology used in the literature to describe where PA occurs within the physical environment (e.g., domain, setting, environment, place, area), an a priori definition of the term location was developed. Locations were defined as natural or built places and areas within a geographic region where individuals spend time during the day and where their activity behavior can be characterized as sedentary, light, moderate or vigorous. They could further be described as: (a) commercial facilities (e.g., shops, entertainment, restaurant); (b) neighborhood/residential areas that include the home (e.g., house, garden, lawn, streets, sidewalks,); (c) recreational facilities and areas (e.g., parks, playgrounds, pools, gym, sports facilities, woodland, lake, beach); (d) schools or workplaces (i.e., school/work building, cafeteria, classroom/office, schoolyard, school gym/sports facilities). A description of a location merely as “outdoors” or “indoors” was not considered as a sufficient description of the location and required further specification with regard to the physical locations described above. The same applied to natural areas or built environments without any further specification and away-from-home areas. In studies that examined PA occurring in domains, such as home, school, transport or leisure, only the data from the home and school domains were considered, because these can be geographically located, whereas the transport or leisure domains do not provide information on spatial context.

### 2.1. Eligibility Criteria

Any study that examined where PA of healthy individuals aged three to 99 years occurred was included. Studies with cross-sectional designs, randomized controlled trials and pre–post studies were included; however, only baseline data were considered. PA had to be examined using device-based tools (e.g., accelerometers), reported in minutes and time-matched with location data. In addition, PA data had to be collected for a minimum of four consecutive days (minimum wear time) [32]. Data on activity locations had to be collected for five consecutive hours per measurement day using either device-based tools (i.e., GPS) or self-report measures, such as questionnaires or logbooks. Studies that only reported the percentage of time where PA occurred as well as studies that employed subjective and qualitative measurement tools of PA were excluded. Moreover, studies examining populations with specific health impairments, including individuals with overweight and obesity or cognitive and psychological disorders, were excluded. Lastly, all included articles had to be published in English language and in peer-reviewed scientific journals.

### 2.2. Information Sources and Search Strategy

The literature search was carried out on 5 August 2020 in the databases Pubmed (Ovid), CINAHL (EBSCO), SPORTDiscus (EBSCO), PsycInfo (EBSCO), and Scopus (Elsevier). A comprehensive search strategy was developed using the SPIDER approach with a combination of keywords in the categories study sample, design, and evaluation [33]. The search formula was as follows: (child* or youth* or adolescen* or boy* or girl* or woman or women or man or men or adult* or elderly or aged) and (questionnaire* or survey* or assessment* or measur* or monitor* or acceleromet* or track* or global positioning system or GPS or geographic information system or GIS) and (location* or environment* or indoor* or outdoor* or space or spaces or place or places) and (physical activ*).

### 2.3. Study Selection

Two reviewers independently screened and selected the relevant articles (A.K. and M.C./Am.K./S.M./J.S.). In the first step, titles and abstracts were scanned, followed by the screening of full-text articles. Any disagreements were discussed between the reviewers and a third researcher (Y.D.) until a consensus was reached. Records were managed in Covidence systematic review software (Veritas Health Innovation, Melbourne, Australia) and EndNote x9 (Clarivate Analytics, Philadelphia, PA, USA).

### 2.4. Data Collection Process

Two reviewers (A.K. and M.C./Am.K./S.M./J.S.) extracted the study information independently using a data extraction form, which was piloted before data extraction onset. The extracted details included general study information, description of the study sample, measurement instruments used for PA and location assessment, description of locations, results on PA and sedentary time (ST) (only in minutes), behavior settings, and risk of bias. Additionally, the studies were evaluated in terms of the degree to which sex and/or gender aspects were considered in the development, conduction, and evaluation of the study using a sex/gender checklist [34].

The extracted locations of PA were categorized into the behavior settings described in the ecological model of active living by Sallis et al. [10]: (1) Neighborhood environment (including streets, roads, pavements, residential area); (2) Recreational environment (e.g., parks, playgrounds, gym, sports facilities); (3) Home environment (including house, backyard, lawn); (4) Workplace environment; (5) School environment (e.g., inside school building and schoolyard). Commercial, shopping and service facilities were grouped as commercial locations. 

Data on PA that occurred in unspecific or miscellaneous locations labelled, amongst others, as “indoors”, “outdoors”, “other”, “outside of area”, “not at home” “nondescript locations”, or “activity locations” were not extracted. If information was missing or the clarification of data was required, the corresponding authors of the included studies were contacted with one contact attempt.

### 2.5. Risk of Bias Assessment in Individual Studies 

The Cochrane Collaboration’s tool for assessing risk of bias in randomized trials [35] was used to assess the methodological quality of each study. Two reviewers assessed the methodological quality independently (A.K. and M.C./S.M./Am.K./J.S.). Any disagreements in judgements were discussed with a third reviewer (Y.D.) until a consensus was reached. A critical assessment of the domains selection bias, performance bias, attrition bias, detection bias, selective reporting, and other bias was performed following the procedure introduced by Prince et al. [27]. Some minor adjustments were made due to the different inclusion criteria of this review. Each entry was rated with a low, high or unclear risk of bias. In detail, selection bias was rated high when authors described that their study sample was a convenience sample, not representative, or selection bias was given. Performance bias was rated high when self-report measures were used to examine locations of PA and low if GPS was used to examine PA locations. Detection bias was rated high if non-validated devices and non-age-specific cut-points were used to describe PA levels. Attrition bias was rated high risk when missing data were >10%. Selective reporting was rated high when the examination of PA locations was not the primary aim of the study but rather the result of secondary data analysis. Other risk of bias was rated high when authors stated that no confounders were examined, or the confounder analysis was not performed appropriately. If the studies did not provide enough information to rate either low or high risk of bias, they were rated unclear.

### 2.6. Sex/Gender Checklist

Recently, a sex/gender checklist was introduced to examine how detailed studies deal with the terms sex (biological construct) and gender (social construct) and how detailed sex/gender differences are considered in the study design, data analysis, and interpretation of data in intervention studies [34]. In accordance with the Cochrane Sex/Gender Methods Group, the terminology “sex/gender” was used to emphasize the entanglement of the constructs sex and gender, when examining possible biological and social differences in PA between men and women, boys and girls and people with diverse gender identities [36,37].

In order to apply this checklist to cross-sectional studies, adjustments were made. The adapted version contained seven items in the categories Background and Concepts (definition and use of sex and/or gender terminology; sex/gender background information regarding the research question); Study Design (validity and reliability of measurement instruments; measurement/selection of PA locations; study sample recruitment); Presentation of findings (statistical results), and Interpretation of findings (discussion). Two review authors (A.K. and K.W.) independently rated each item based on the amount of information provided with either no information provided, or basic, detailed, or not relevant. Only one subcategory (definition and use of sex and/or gender terminology) could be rated as poor if the terms sex and gender were used interchangeably.

### 2.7. Summary Measures and Synthesis of Results

Due to the heterogeneity of the studies with regard to the measurement methods, the classification of PA locations, and the approaches used to analyze PA data (e.g., differences in measurement devices, wear time, cut-points used to define PA levels), the extracted data were summarized narratively. Specifically, the mean (or median) number of minutes engaged in PA, including standard deviations (SD) or interquartile range (IQR), were summarized narratively for each identified location and behavior setting. Furthermore, differences between male and female participants were examined when gender-specific PA data was provided; *p*-values ≤ 0.05 were considered statistically significant. Studies were categorized into two groups according to the stage of life of the examined study samples: (1) children and adolescents (3 to 18 years of age); (2) adults (19 years and older) [38]. Table 1 presents all studies conducted in children and adolescents and Table 2 presents all studies conducted in adults.

## 3. Results

### 3.1. Study Selection

**Figure 1:**
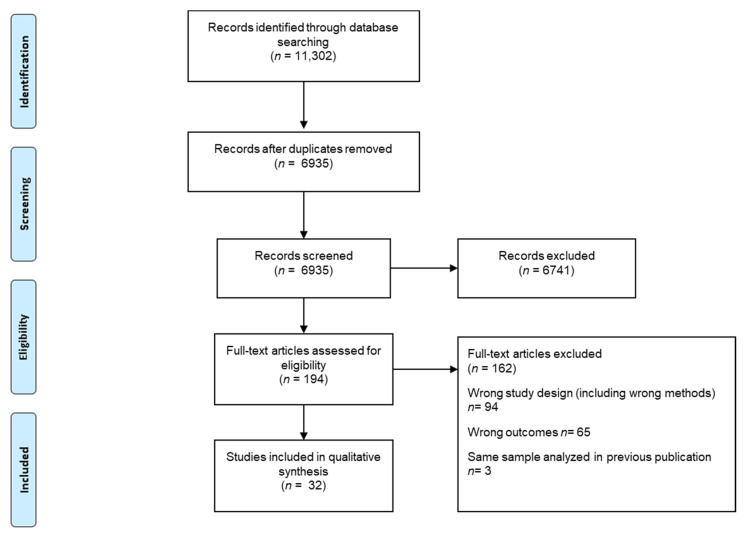
Flowchart of the identification of included studies.

The database searches identified 6935 publications without duplicates. Initially, 35 studies were considered eligible for inclusion. However, after completion of the full-text screening, three articles were removed because they contained data that had been published in previous manuscripts that had already been included [39,40,41]. In total, 32 studies were included for data extraction and methodological quality assessment (Figure 1).

### 3.2. Study Characteristics

The studies were conducted in the USA (*n =* 14); UK (*n =* 6); the Netherlands (*n =* 5), Switzerland (*n =* 2); Brazil (*n =* 1); Canada (*n =* 1); Denmark (*n =* 1); Spain (*n =* 1); and also included a Trans-European study (Spain, UK, Netherlands, Lithuania), published between 2009 and 2019. Among these studies, 22 studies examined locations of PA in school-aged children and adolescents (Table 1) [42,43,44,45,46,47,48,49,50,51,52,53,54,55,56,57,58,59,60,61,62,63] and ten studies examined the locations of PA in adults (Table 2) [64,65,66,67,68,69,70,71,72,73]. Two of the adult studies explicitly examined older adults [68,70]. The lowest age of participants was 8.5 (SD = 0.3) years [45], the highest was 81.1 years [70]. Study sample sizes ranged from 24 [57] to 1053 [63]. In total, 30 studies included both male and female participants. Of these, ten studies reported separate results for male and female participants [44,47,49,51,52,53,60,63,70]. Significant differences between sex/gender groups are indicated in Table 1 and Table 2 when applicable.

In regard to the measurement instruments, ActiGraph accelerometers (Pensacola, Florida, USA) were the most widely used devices (*n =* 29). Three studies used PA monitoring devices such as heart rate monitoring [47], the Actical accelerometer [71] or the smartphone application CalFit [73]. The data collection period of PA (wear time) ranged from four days [51] to 33 days [55]. Different PA outcomes as well as sedentary time (ST) were reported across the studies: ST (*n =* 10); light PA (*n =* 7); moderate PA (*n =* 4); vigorous PA (*n =* 6); moderate-to-vigorous PA (MVPA, *n =* 27). Three adult studies examined minutes of PA (including bouts) without classifying PA into its different intensities [64,70,72]. Only one study implemented a self-report measurement tool (in combination with Google Maps) to identify the locations of PA, which were time-matched with PA data [53]. All other studies (*n =* 31) examined the locations of PA using some type of GPS system (e.g., Qstarz BT-Q1000X GPS or Garmin Forerunner GPS) in combination with GIS (*n =* 27) and/or Google Maps (*n =* 3).

### 3.3. Risk of Bias within Studies

**Figure 2:**
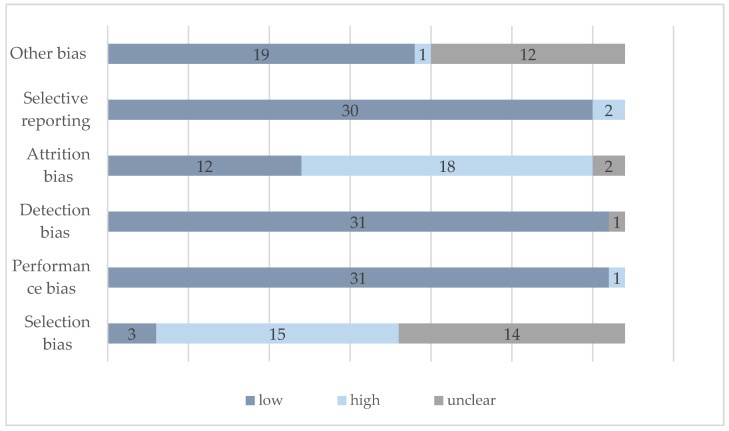
Risk of bias of included studies.

An overview of the risk of bias for all studies is given in Figure 2. The study by Bürgi et al. [44] had a low risk of bias in all six categories. Across the included studies, the lowest risk of bias was found in the domain performance bias, detection bias, and selective reporting. In the domain attrition bias, most studies (*n =* 18) were rated with a high risk of bias due to data loss >10%. Nearly half of the studies were rated with a high risk of selection bias (*n =* 15), whereas 14 studies did not provide sufficient information on this item and the risk of bias was unclear.

### 3.4. Sex/Gender Checklist

**Figure 3:**
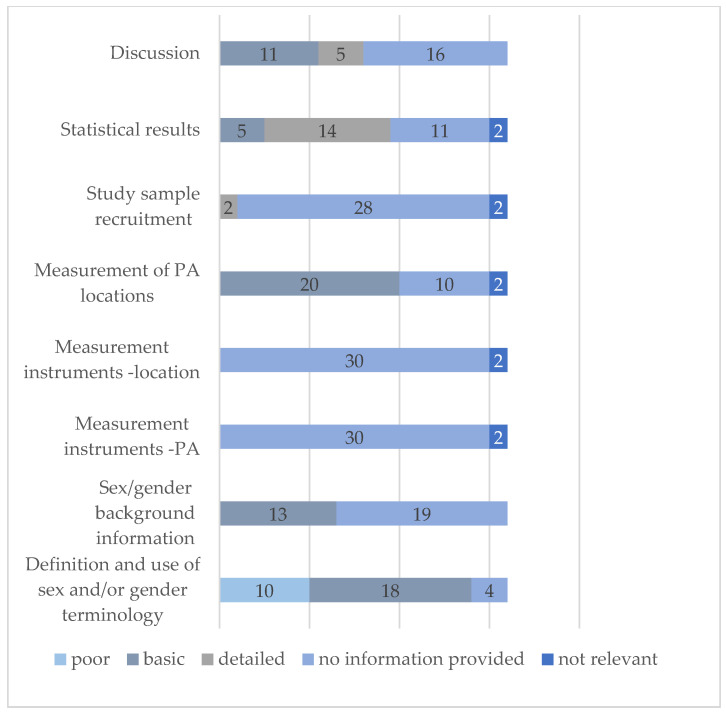
Analysis of the sex/gender checklist across studies. Note. Abbreviation: PA, physical activity.

Figure 3 shows the evaluation of the sex/gender checklist across the included studies. Over half of the studies used the terms gender or sex consistently throughout their article (*n =* 18), whereas ten studies used these terms interchangeably and were rated poor. More than half of the studies (*n =* 19) did not consider sex/gender while providing background information for their research question and almost no study provided information on the reliability and validity of the applied measurement instruments for different sex/gender groups (*n =* 30). In the single sex/gender studies, this item was not considered relevant. Twenty studies did not preselect the examined locations of PA and provided basic information for considering sex/gender differences in the statistical analysis. Of the 32 studies, two studies provided detailed information on their sampling methods and aimed at a balanced sample of males and females [46,70]. The majority of studies provided information on the statistical analyses for sex/gender differences: five studies provided basic information and 14 studies provided detailed information. About half of the studies reflected their results with respect to sex/gender differences (*n =* 11) and discussed future directions for sex/gender interventions (*n =* 5).

### 3.5. Synthesis of Results 

In the following, the results of individual studies on the location of PA and the allocated behavior settings across the lifespan are reported and summarized separately by age group.

#### 3.5.1. Locations of PA and Behavior Settings in Children and Adolescents

In children and adolescents, PA accumulated in the neighborhood, school, and recreational environment were examined most frequently (each *n =* 14). The locations within the neighborhood behavior setting included, amongst others streets, roads and pavements [44,45,47,51,54,56,57,58], residential vegetated and built land use areas [55], and residential or neighborhood areas with buffer zones around the geocoded home ranging from 500 m [43] to 1 km [46,48,51,53]. One study compared PA in youth-defined neighborhoods and census-defined neighborhoods [61].

The studies that examined PA occurring in the school environment specified the locations as: (own) school [42,44,45,46,52,53,56,57,58,59], inside school building [49], schoolyard [49,62], school grounds [52,60], school grounds green space [54], near school [46], and other schools (where children or adolescents were not enrolled in) [44,45,53]. Buffers around these locations ranged from 10 m [44,45,49,52,60] to 1 km for the near school area [46]. One study further examined PA occurring in afterschool childcare centers [60].

Diverse locations within the recreational environment were examined and labelled amongst others as parks or playgrounds [45,50,51,52,53,54,55,57,58], sports/recreational facilities [44,45,52,60], and greenspace areas [52,54,56,63], including gardens [51,54], farmland/agriculture [51,55], and woodland or beaches [51]. Kneeshaw-Price et al. [53] further differentiated between “public, outdoor parks, recreational facilities” and “private recreational facilities, public indoor recreational facilities”.

Physical activity occurring at home (home environment) was examined in twelve studies and home buffers ranged from 10 [42,52] to 50 m [46] around the geocoded home address/home perimeters.

In a few studies (*n =* 4), PA was also examined in commercial facilities, including shops, shopping centers, service locations, and food eateries [52,53,55,60]. The study by [55] examined PA in commercial vegetated and commercially built land-use types. 

In addition to identifying locations of PA, our aim was to examine the activity behavior within these locations, i.e., minutes of PA and ST. Different activity levels were reported across the studies, ranging from light to vigorous PA. Most often, minutes of MVPA were reported either per day, per week or summarized across varying observation days. For reasons of comparison, we focused on those studies reporting minutes of MVPA per day (*n =* 14) [42,43,46,47,48,49,52,53,54,58,60,61,62,63] and minutes of daily ST (*n =* 5) [42,48,54,58,62].

Mean minutes of MVPA in neighborhood locations ranged from 2.1 (SD = 6.5) minutes per day [53] to nearly 33 minutes on weekend days (mean minutes of MPA and vigorous PA combined) [48]. Specifically on streets, daily minutes of MVPA ranged from 1.9 (SD = 3.2) minutes [54] to 16.93 (SD = 18.44) minutes per day [47]. The duration of MVPA in the school environment ranged from 0.1 (SD = 3.3) minutes [54] to 37.6 (SD = 27.4) minutes per day [53]. A more detailed analysis of PA in the school environment showed that the amount of MVPA was higher outdoors on the schoolyard than inside the school building [49]. Klinker et al. [52] and Van Kann et al. [62] examined children’s PA levels on the school grounds during and after the school day and reported more minutes of MVPA during the regular school day than outside school hours. The daily amount of MVPA in recreational facilities in general reached up to 56.40 (SD = 72.83) minutes per day [47]. In particular, MVPA on sports grounds reached up to 41 median minutes per day [60], whereas in parks MVPA ranged from 0.7 (SD = 4.7) mean minutes per day (weekdays) [54] to 6.9 (SD = 14.9) mean minutes per day [53]. At home, children’s minutes of MVPA ranged from 4 (IQR = 2−8) median minutes per day [58] to 62.6 (SD = 36.7) mean minutes per day [53]. Time in MVPA accumulated in commercial locations ranged from 0.0 median minutes per day [52] to about 8.0 min per day [53,60].

Two studies examined daily ST in neighborhood locations ranging from 2.8 (SD = 7.2) mean minutes [54] to 143.3, 95%CI [137.9−148.8] mean minutes on weekdays [48] and 3.9 (SD = 12.5) mean minutes [54] to 334.2, 95%CI [321.6−346.9] mean minutes on weekend days [48]. At home, ST ranged from 50 (IQR = 40−69) median minutes per day [58] to 181.6 (IQR = 48.3−286.2) median minutes per day on weekend days [42]. In the school environment, ST ranged from 20.16 (SD = 12.0) mean minutes per day accumulated outdoors on the schoolyard [62] to 87 (IQR = 63−110) median minutes per day at school in general [58]. In recreational locations, such as parks, the mean daily ST ranged from 1.1 (SD = 6.8) minutes on weekdays to 3.4 (SD = 19.1) minutes on weekends [54].

In total, nine of the 21 studies provided sex/gender-specific results of location-based PA in minutes per day or overall [44,47,49,51,52,53,60,63]. Of these, six reported significant between-group differences in location-based MVPA [44,51,52,53,60,62]. Kneeshaw-Price et al. [53] further examined sex/ gender differences between age groups. Although no descriptive statistics were provided by Almanza et al. [43] and Carlson et al. [46], and Remmers et al. [60], these studies confirmed sex/gender differences in minutes of MVPA by location. Overall, (younger) boys accumulated more minutes of MVPA than (older) girls in the school environment [44,46,52,53,60,62], recreational environment (e.g., in sports facilities, parks, playgrounds, green spaces) [52,53,60], and neighborhood environment [43,51]. At the home location, two studies reported fewer minutes of MVPA for (older) girls than for (younger) boys [46,53], whereas one study found that girls engaged in more minutes of MVPA than boys [60]. Younger girls were also found to accumulate more minutes of MVPA in shopping locations than older boys, but older girls accrued significantly less MVPA in service locations than younger boys [53]. Jones et al. [51] further reported significant sex/gender differences in location-based MVPA bouts in relation to the land-use type; however, these were not found in the average summarized four-day MVPA minutes. Additionally, Van Kann et al. [62] reported more minutes of ST on the schoolyard in girls during morning recess than boys.

#### 3.5.2. Locations of PA and Behavior Settings in Adults 

In adults and older adults, the most frequently examined locations of PA were the home [64,66,67,68,69,71] and recreational environment [65,66,69,70,72,73] (*n* = 6). Buffers around the geocoded home ranged from 25 m [69] to 536 m [71]. Locations in the recreational environment included parks [65,66,72], green spaces [69,70,73], or sport facilities [66,69]. PA in the neighborhood environment was examined in three studies and included locations such as streets, footpaths/trails [66] and neighborhood or residential areas and facilities [66,67,69]. Buffer zones for neighborhood and residential areas ranged from 25 [69] to 1166 m [67]. Only one study examined PA occurring in workplaces (working environment) [69] and one study examined PA in schools (school environment) [66]. Commercial locations were examined in two studies [66,69]. 

Similar to the studies in children and adolescents, different PA levels were examined ranging from light PA to vigorous PA and ST. Most often, minutes spent in MVPA were reported per day [65,67,68,69,71,73] followed by minutes of MVPA bouts per day [64,72] and total minutes of MVPA bouts over three weeks [66]. Again, we focused on studies reporting daily minutes of MVPA and ST.

Minutes of MVPA at home ranged from 0.1 (IQR = 0.02–0.27) [68] median minutes per day to 10.4 (IQR = 16.8) median minutes per day [69]. Within the recreational environment, Evenson et al. [65] reported 0.5 (IQR = 0.0–2.7) median minutes of MVPA per day accumulated in parks. Green spaces accounted for 0.9 (IQR = 6.6) median daily minutes of MVPA [69] to 7.75 (IQR = 24.12) [73] median minutes of MVPA on weekend days; sports facilities accounted for up to 4.2 (IQR = 19.6) median minutes of MVPA per day [69]. In the neighborhood environment, minutes of MVPA ranged from 0.6 (IQR = 2.8) median minutes per day [69] to about 20 mean minutes per day [67]. At the workplace, 9.9 min (IQR = 19.6) of MVPA were accumulated per day [69], whereas in commercial locations, 1.0 (IQR = 4.1) minute of daily MVPA was accumulated in shopping areas [69].

Daily ST was examined in three studies. Hurvitz et al. [67] and Jansen et al. [68] found that mean minutes of ST at home ranged from 183.3 (SD = 90.7) minutes per day to 370.32 (SD = 159.61) minutes per day. Evenson et al. [65] reported that adults spent 3.8 (IQR= 0.6–11.0) median minutes per day being sedentary in parks.

Of the included studies conducted in adults, only one study reported sex/gender-specific results of location-based minutes of PA [70]: median sedentary and active time in urban green spaces did not significantly differ between male and female senior residents.

## 4. Discussion

This systematic review included 32 studies that examined the locations of PA and sedentary behavior in school-aged children, adolescents and adults. A variety of PA locations were identified and allocated to the neighborhood, recreational, home, school, or workplace environment, introduced as key behavior settings of active living domains by Sallis et al. [10]. Additionally, commercial locations (e.g., shopping, food locations) were identified as contributors to daily PA from childhood to adulthood. 

### 4.1. Locations of PA in Children and Adolescents

The majority of reviewed studies (*n =* 22) examined children and adolescents’ PA levels on a daily basis and most commonly reported PA occurring in the neighborhood, school, recreational (each *n =* 14), and home environment (*n =* 12) followed by commercial facilities (*n =* 4). Locations in the home [53] and recreational environment [47] provided the highest mean values of MVPA per day. However, taking a closer look at daily minutes of MVPA accumulated at individual locations within the recreational environment showed that mean minutes spent in MVPA in parks and green spaces were quite low; two studies reported less than three minutes of daily MVPA accumulated in parks [54,58]. The school environment was a greater source of daily MVPA than the neighborhood environment, yet less of a daily source of MVPA than the home or recreational locations, when comparing the highest mean values between these locations [47,48,53].

Depending on the land-use types, several studies reported higher minutes of MVPA (daily or overall) accumulated in built locations, such as buildings, streets and pavements than in locations with vegetated/green spaces [45,52,53,54,55,56,57,58,63]. Only Jones et al. [51] reported higher minutes of MVPA in gardens than in buildings and roads, pavements. These findings could be attributed to the fact that children and adolescents spend more time in built locations, enabling them to accrue more minutes of PA in these land-use types. However, we did not examine this.

Coincidently, the neighborhood [48], home [42,58] and school [42,58,62] environments were large sources of daily ST. Higher ST was found at home and in the neighborhood on weekend days compared to weekdays [42,48], whereas the lowest daily ST was reported in outdoor recreational locations (parks, green spaces) and on the schoolyard [54,62]. However, the amount of ST in the latter locations was higher than the amount of MVPA [54,62]. The same applies to the home and in neighborhood locations [42,48,58].

These findings are in line with previous location-based reviews that identified homes and school grounds as important locations for MVPA in children and adolescents [23,27]. Both reviews found that streets and built land-use types were major sources of MVPA, whereas green areas and parks were rather low sources of total PA. However, this holds true only for the absolute minutes of PA and not the proportion of total time spent in these locations, which in turn could be quite high [23,27].

A possible explanation for higher MVPA values reported on streets, roads and pavements could be their use for active transportation to school or other locations in the neighborhood environment. Examining associations between environmental attributes and PA, Davison and Lawson [11] found that children were more active when transport infrastructure, such as sidewalks, in the neighborhood, destinations to walk to, controlled intersections, or absence of road hazards, was provided. Furthermore, the availability of recreational facilities in the neighborhood, equipment and permanent activity structures in school play areas were associated with higher PA. They found no evidence for the association between home equipment and children’s PA [11].

Sex/gender differences in PA by location were reported in each behavior setting: home, neighborhood, school, and recreational environment. Boys were consistently reported to accumulate more minutes of MVPA in each of these behavior settings, except for the home environment, where one [60] study found that girls engaged in significantly more minutes of MVPA than boys did. These results may imply that girls engage in less PA, independent of the PA location. A previous study on gender differences in domain-based PA showed similar results: boys’ proportion of MVPA time in the home and school environment was significantly higher compared to girls [74]. To increase girls’ PA and to mitigate sex/gender differences in location-based PA, more knowledge about sex/gender barriers of location-specific PA is needed. The study by Pawlowski et al. [75], for example, revealed that boys and girls identified the same barriers (e.g., weather, lack of space or play facilities) for PA during school recess, but intra- and inter-gender differences in the perceptions of barriers were present.

In summary, we identified locations that were more or less associated with children and adolescents’ daily PA. To increase daily PA in recreational locations, such as parks, playgrounds and other green areas, city planners should aim to create activity-friendly design features that encourage children and adolescents to become physically active, taking sex/gender differences into account. This also applies to school play areas. Accessibility to recreational PA locations, which are commonly in close vicinity to the home and within the neighborhood environment, must be supported by providing transport infrastructure that is pedestrian and cycling friendly. Lastly, the home location emerged as an important source for daily PA, despite the simultaneously reported high sedentary time. Future research should examine which physical attributes in the home environment can promote PA in boys and girls equally.

### 4.2. Locations of PA in Adults 

In adults, frequently examined locations of daily PA included the home (*n =* 6), recreational (*n =* 6), and neighborhood environments (*n =* 3). Commercial locations (*n =* 2) and the workplace (*n =* 1) were examined less frequently. The highest average amount of MVPA was found in neighborhood locations with up to approximately 20 minutes of MVPA per day [67], followed by the home [67,69], workplace [69], and recreational locations, such as green spaces [73]. Similar findings were reported in one of the included studies that examined PA over a period of three weeks [66]. Adults accrued most bout minutes of MVPA at home, in the neighborhood-built environment (on roads), and in recreational environments (parks). Commercial facilities provided more bout minutes of MVPA than other residential areas, footpaths, and trails [66]. This suggests that the locations used for PA and the activity patterns within these locations might be stable over time.

Daily minutes of ST exceeded those of MVPA in parks [65], in the neighborhood [67] and at home [67,68]. The least amount of daily ST by location was reported in parks [65], which could be explained by the total time spent in the individual location increasing from parks to the home location, though we did not examine total time spent in the locations of interest.

These results are in accordance with the previous review conducted by Prince et al. [27], who found that most MVPA occurred outside the home area. However, in contrast to Prince et al. [27], the results of this review showed that the home location was an important source of daily PA and accounted for similar minutes of MVPA as recreational and workplace locations. This could be attributed to the studies under investigation: seven out of nine adult studies included individuals over 65 years [65,66,67,68,70,72,73], which may spend the majority of their day at home rather than at work or in transport-related activities on streets and roads within the neighborhood. Merely one study examined PA occurring at the workplace [69], yet the minutes of MVPA per day reported were consistent with a previous review and meta-analysis on PA, ST and cardio-metabolic health and fitness among diverse occupational groups [76]. While approximately ten minutes per day were spent in MVPA at work, the majority of daily worktime consisted of sedentary behaviors [76].

Of the examined studies, only one provided a sex/gender-specific analysis of minutes of PA by location in adults. In this case, senior residents’ minutes of PA did not differ between males and females [70]. Based on this one study, no conclusion can be drawn as to which locations are more or less used for PA by male or female adults.

In summary, the home and neighborhood environment emerged as important sources of daily PA in adults. Previous research has shown that environmental attributes, such as the presence of PA facilities, sidewalks, shops, services and not perceiving traffic as a problem, are positively associated with adults’ PA [13]. Karmeniemi et al. [77] further concluded that diverse residential areas in which housing is mixed with commercial, public, and recreational locations within walking and cycling distance can promote daily PA.

A systematic approach to PA promotion should consider the significance of these environmental structures. City planners must focus on developing highly connected neighborhoods, especially where older adults have access to nearby recreational and commercial locations. In addition, further research is needed into the physical characteristics of the home and workplace environment that encourage adults to engage in more PA.

### 4.3. Methodological Quality and Limitations of Primary Studies

The primary studies included in this review have limitations that affect the overall validity of this review. Nearly half of the included studies analyzed data from convenience samples, limiting the generalizability of their findings. Although some studies had large sample sizes (*n* > 500) [46,48,53,63], it cannot be assumed that the samples were representative of the population under investigation. Nearly all studies had a low risk of performance and detection bias because they used a GPS device to track the locations combined with a device-based measurement of PA using established cut-points to estimate time spent in various PA levels as well as in sedentary behaviors. However, over half of the studies reported a high risk of attrition due to a data loss of >10%.

Despite the well-known differences in PA between males and females across the lifespan [1], only nine of the included studies, conducted foremost in children and adolescents (*n =* 8), reported descriptive PA data (in minutes) for male and female participants separately. Over half of these studies confirmed differences in minutes of PA in diverse PA locations between boys and girls [44,51,52,53,60,62]. The neglect of sex/gender, as an important intra-personal determinant of active living [10], is partly reflected in the results of the sex/gender checklist. The majority of studies (*n =* 19) did not provide information on how sex/gender differences were considered in the research question or study design, yet sex/gender differences were later considered in the statistical analyses. Future research, especially studies conducted in adults, should try to gain a deeper insight into the differences in the locations used for daily PA between males and females in order to develop successful sex/gender-specific intervention strategies.

### 4.4. Strengths and Limitations of this Review

The current systematic review has some strengths and limitations that should be taken into account when interpreting and evaluating the results. The strengths of this review lie in (1) the thorough and systematic search of relevant studies; (2) evaluating the methodological quality (risk of bias) of included studies; (3) analyzing the extent to which sex/gender was considered in the reviewed studies; (4) focusing on specific, geographically identifiable locations where PA may take place; (5) focusing on minutes (per day) engaged in PA in specific PA locations.

However, differences in the approaches used to examine location-based PA, as well as differences in the reporting of data and classification of locations, hinder a comprehensive comparison of the reviewed studies. In detail, the included studies had different inclusion and exclusion criteria for their location and PA data [22]. The minimum wear time of PA devices per day, total included days, wear-time-validation, non-wear-time definition and PA cut-points varied strongly across the studies. Not only could these methodological decisions affect attrition rates, but they also limit the comparability of findings across studies. One explicit example is the study by [66], which examined and compared PA bout minutes in the same PA locations using two different cut-points for MVPA, Matthew’s MVPA ≥ 760 counts per minute and NHANES MVPA ≥ 2020 counts per minute. MVPA bout minutes varied largely between the two cut-points [66]. In addition, the included studies were heterogeneous in the reporting of their results ranging from minutes per day, bout minutes per day, weekly minutes, to total minutes across observation days (e.g., four days to three weeks). Reporting mean activity counts per minute instead of converting counts to activity levels could be one step to help improve the comparability of PA data and minimize errors in the assumptions that are made based on different cut-points. It would be desirable to report raw data processing and filtering to outcome metrics for an across device-comparison [28]. Moreover, some studies aimed at a full-day approach to examine PA locations, others focused on PA occurring in specific locations for a limited time of the day.

To date, there is no standard approach for the interpretation and analysis of GPS data [23,24] and the use of GPS is not without limitations. Data loss can be attributed to heavy tree cover or being inside buildings [24]. Using multiple devices (i.e., GPS and accelerometer) requires the integration of GPS and PA data, which again demands multiple decisions on data processing. Most studies in this review (*n =* 31) combined GPS and PA data to locate PA and provide contextual information to PA and sedentary behavior, yet the approaches used to match these two types of data varied across the studies. The inclusion, removal and classification of GPS data points also differed. For example, Lachowycz et al. [54] and Wheeler et al. [63] assumed that accelerometer time with missing GPS data points were due to lost signals and classified this as being indoors. Other studies excluded data points at certain speeds because they were associated with motorized travel or rated as erroneous measurements of the GPS device, e.g., [43,50,54]. Furthermore, there is no standard in categorizing GPS data points into locations of interest. This is amplified by using different large buffer zones for the same locations, which are used to account for potential errors in GPS data. For example, buffers around the home varied from 10 to 536 m [52,71]. All of these methodological decisions may lead to misclassification and misinterpretation of data [23,24,27].

Lastly, this review only included healthy individuals, which limits the generalizability of findings to specific groups of individuals that are at increased risk for insufficient PA, including overweight and obese individuals. Further research is needed to identify important locations of PA for specific subgroups.

## 5. Conclusions

The findings of this review indicate that different physical locations are used for PA to a varying degree over the lifespan. In childhood and adolescence, the home, recreational and school environments are important sources of daily PA. Especially, built locations, including buildings, streets, and pavements, provide children and adolescents opportunities for being physically active. In contrast, less time is spent being physically active in parks and green spaces. In adults, the home and neighborhood environment are valuable sources of daily PA, followed by recreational facilities, including parks and green spaces. Of the reviewed studies in adults, only one provided sex/gender-specific analyses of location-based PA, indicating a need for further investigation.

These findings have implications for the promotion of PA from childhood to adulthood as they highlight the importance of the home and neighborhood environment for daily PA. City planners should make it their top priority to develop highly connected neighborhoods that are safe to walk and bike within, as outlined in the World Health Organization Global Action Plan on Physical Activity [78]. In order to create healthy environments in all domains of active living, further research is needed to find out which strategies and physical characteristics can promote daily PA in the home, at the workplace and in recreational locations for males and females alike.

## Figures and Tables

**Table 1:** Description of included studies targeting children and adolescents and summary of main findings.

Study Details	Participant Characteristics	Measurement Tools and Days of Data Collection	Physical Activity Cut-Points	Description of Locations	Minutes of Physical Activity in Identified Locations	Behavior Setting
Alberico et al., 2017, Brazil [42]	*n* = 80, *n*_male_ = 46, *n*_female_ = 34 Age = 12 to 17 years; *M* = 14.5 (SD = 5.5) years	QStarz BT-1000X and BT-1000XT GPS, ActiGraph GT3X and GT3X+, 7 days	ST ≤ 100 cpm, MVPA ≥ 2296 cpm	Home (10 m buffer), School (geocoded school’s parcels)	Median minutes (IQR) daily MVPA and ST on weekdays and weekend days Home_Weekday_ (*n* = 79): MVPA = 5.0 (2.3–8.2); ST = 167.5 (79.8–238.0) School_Weekday_ (*n* = 79): MVPA = 2.0 (0.6–4.9); ST = 63.5 (19.5–132.3) Home_Weekend day_ (*n* = 77): MVPA = 6.6 (1.0–10.0); ST = 181.6 (48.3–286.2)	(3;5)
Almanza et al., 2012, USA [43]	*n* = 208, *n*_male_ = 100 *n*_female_ = 108, *n*_SmartGrowth_ = 65 (*n*_male_ = 30; *n*_female_ = 35), *n*_CG_ = 143 (*n*_male_ = 70; *n*_female_ = 73) Age = 8 to 14 years	GlobalSat BT-335 GPS, ActiGraph GT2M, 7 days	MVPA ≥ 4 MET (Freedson equation: MET = 2.757 + (0.00 15 x cpm)−(0.08957 x age (year))−(0.000038 x cpm x age (year))	Neighborhood (within 500 m of the home, excluding home buffer (30 m buffer around home))	Median daily minutes of MVPA (range) Smart Growth Community Neighborhood (*n* = 65) = 7.50 (0–36.50) Conventional Community Neighborhood (*n* = 143) = 4.25 (0–47.67) Boys engaged in 1.58 times the daily rate of neighborhood-MVPA of girls, *p*-value < 0.01.	(1)
Bürgi et al., 2015, Switzerland [44]	*n* = 119, *n*_male_ = 51, *n*_female_ = 68 Age = 6th grade; *M* = 12.5 (SD = 0.4) years	QStarz BT-Q1000XT GPS, Actigraph GT3X, 7 days	MVPA ≥ 2296 cpm	Home (30 m buffer), Own school (main school where student goes to school, 10 m buffer), Other school (school grounds of all public schools in Winterthur, except own school, 10 m buffer), Recreational facility (public parks and sport facilities in Winterthur, 10 m buffer), Street (streets, sidewalks, cycle or pedestrian paths in Winterthur, 10 m buffer)	Weekly median minutes of MVPA (IQR) Home: Total = 34.0 (18.5–59.0); Boys = 41.5 (20.2–60.2); Girls = 30.8 (17.3–55.3) Own school: Total = 74.7 (51.2–108.3); Boys = 80.3 (58.2–136.7); Girls = 71.9 (40.1–91.8) Other school: Total = 3.7 (0.3–29.0); Boys = 14.5 (0.2–45.8); Girls = 3.3 (0.8–24.3)* Recreational facility: Total = 4.7 (0.3–19.8); Boys = 9.2 (1.2–24.7); Girls = 2.7 (0.3–13.3) Street: Total = 94.3 (57.0–143.7); Boys = 91.0 (54.8–145.0); Girls = 99.0 (65.2–139.5) Significant difference between boys and girls at *p < 0.05	(1;2;3;5)
Bürgi et al., 2016, Switzerland [45]	*n* = 83, *n*_male_ = 43, *n*_female_ = 40 *n*_low SES_ = 38, *n*_high SES_ = 45 Age = 2nd grade classes; *M* = 8.5 (SD = 0.3) years	QStarz BT-Q1000XT GPS, Actigraph GT3X, 7 days	ST < 101 cpm MVPA ≥ 2296 cpm	Home (30 m buffer), Own school (school grounds of main school where student goes to school, 10 m buffer), Other school (school grounds of all public schools in Zurich, except own school, 1 m buffer), Park (public parks and playgrounds in Zurich, 10 m buffer), Sport (sport facilities in Zurich, 10 m buffer), Street (streets, sidewalks, cycle or pedestrian paths in Zurich, 10 m buffer)	Median weekly minutes (IQR) of MVPA Home: Total = 57.3 (32.2–91.8); low SES = 66.3 (38.7–105.3); high SES = 47.2 (31.5–76.7) Own school: Total = 121.5 (86.2–184.3); low SES = 125.2 (90.7–189.2); high SES = 118.3 (85.3–163.2) Other school: Total = 13.0 (3.3–28.2); low SES = 10.8 (4.8–22.0); high SES = 16.2 (3.2–33.2) Park: Total = 9.3 (1.5–29.5); low SES = 3.3 (0.5 -12.8); high SES = 18.2 (5.3–48.7) Sport: Total = 4.3 (0.3–21.3); low SES = 1.1 (0.3–22.5); high SES = 6.7 (0.3–20.3) Street: Total = 90.5 (56.0–127.0); low SES = 69.3 (42.7–111.2); high SES = 102.8 (72.0–135.7) Median weekly minutes (IQR) of ST Home: Total = 529.7 (255.0–798.5); low SES = 538.3 (333.0–683.3); high SES = 470.3 (226.8–846.5) Own school: Total = 597.7 (509.0–731.7); low SES = 584.1 (477.2–724.7); high SES = 600.2 (533.3–749.2) Other school: Total = 46.7 (4.7–87.3); low SES = 45.8 (25.2–82.3); high SES = 46.7 (2.0–89.2) Park: Total = 15.7 (1.7–57.8); low SES = 6.0 (0.0–24.3); high SES = 47.7 (8.7- 76.8) Sport: Total = 8.5 (0.0 – 52.0); low SES = 7.1 (0.0–46.2); high SES = 10.2 (0.0–70.0) Street: Total = 234.5 (173.3–378.2); low SES = 193.9 (123.2–313.5); high SES = 304.3 (207.5–397.0)	(1;2;3;5)
Carlson et al., 2016, USA [46]	*n* = 549, 49.9% female Age = 12 to 16 years; *M* = 14.1 (SD = 1.4) years	GlobalSat DG-100 GPS, ActiGraph (Models 7164, 71,256, GT1M, GT3X), 7 days	MVPA ≥ 1148 counts per 30-s epoch	Home (50 m buffer), Near home (1 km street network buffer around home point, excluding at home circular buffer), At school (15 m buffer around school parcel), Near school (1 km street network buffer around school point, excluding at school parcel buffer)	Mean daily minutes of MVPA (SD) At home: Weighted week = 7.4 (7.4); Non-school days = 12.0 (14.1); School days = 5.5 (6.6) Near Home: Weighted week = 5.9 (9.0); Non-school days = 6.8 (11.6); School days = 5.4 (9.2) At school: Weighted week = 16.7 (10.9); Non-school days = 0.6 (11.6); School days = 23.2 (15.0) Near School: Weighted week = 2.2 (3.8); Non-school days = 1.7 (4.9); School days = 2.4 (4.3) Note: Weighted week = ([mean daily minutes across school daysx 5]+[mean daily minutes across non-school days x 2]÷7) Girls engaged in less MVPA than boys: −3.7 min/day at home (*p* < 0.001); −2.6 min/day near home (*p* = 0.001); −5.5 min/day at school (*p* < 0.001).	(1;3;5)
Collins et al., 2012, UK [47]	*n* = 44, *n*_male_ = 19, *n*_female_ = 25 Age = 13 to 14 years	Garmin Forerunner 305 GPS, Heart rate monitor connected to Garmin watch, 7 days (out-of-school hours)	MVPA = 140–159 bpm VPA > 160 bpm	Public recreational facilities (parks, public playgrounds, leisure centers, school playing fields and country parks, shops, restaurants, cinemas, theatre, garden, woodland and extra-curricular education classes), House, Street	Mean daily weekday minutes of MVPA (SD) Suburban youth Public recreational facilities: Male = 56.40 (72.83); Female = 30.22 (56.31) House: Male = 8.28 (10.57); Female = 18.27 (23.35) Street: Male = 15.48 (11.29); Female = 16.93 (18.44) Rural youth Public recreational facilities: Male = 18.88 (61.65); Female = 11.22 (31.89) House: Male = 8.35 (14.14); Female = 9.78 (51.91) Street: Male = 12.77 (37.77); Female = 6.13 (8.83) Mean daily weekday minutes of VPA (SD) Suburban youth Public recreational facilities: Male = 11.51 (24.08); Female = 4.86 (12.41) House: Male = 0.07 (0.19); Female = 0.74 (4.25) Street: Male = 0.63 (1.26); Female = 0.68 (1.48) Rural youth Public recreational facilities: Male = 1.03 (2.6); Female = 1.35 (4.94) House: Male = 0.32 (0.81); Female = 0.39 (1.11) Street: Male = 0.99 (4.99); Female = 0.14 (0.32)	(1;2;3)
Coombes et al., 2017, UK [48]	*n* = 967, *n*_male_ = 413; *n*_female_ = 554 Age = 13 to 15 years; *M* = 13.5 years	Qstarz BT1000XT GPS, ActiGraph GT3X+, 7 days	ST ≤ 100 cpm LPA = 101–2295 cpm MPA = 2296–4011 cpm VPA ≥ 4012 cpm	Home neighborhoods (area within a 10-min walk (equivalent to 800 m) around the home address of each participant)	Mean [95% CI] daily minutes of PA on weekday evenings in home neighborhoods ST: More PA supportive (*n* = 484) = 122.2 [116.6 - 127.7]; Less PA supportive (*n* = 483) = 143.3 [137.9–148.8] LPA: More PA supportive = 39.1 [37.3–41.0]; Less PA supportive = 45.0 [43.1–46.9] MPA: More PA supportive = 8.5 [7.9–9.1]; Less PA supportive = 8.0 [7.4–8.5] VPA: More PA supportive = 5.0 [4.5–5.6]; Less PA supportive = 5.2 [4.7–5.8] Mean daily minutes of PA [95% CI] on weekends in home neighborhoods ST: More PA supportive (*n* = 445) = 290.0 [276.3–303.7]; Less PA supportive (*n* = 438) = 334.2 [321.6–346.9] LPA: More PA supportive = 96.4 [91.4–101.3]; Less PA supportive = 118.6 [113.5–123.8] MPA: More PA supportive = 18.6 [17.0–20.1]; Less PA supportive = 20.5 [18.9–22.0] VPA: More PA supportive = 10.3 [8.9–11.7]; Less PA supportive = 11.9 [10.4 -13.4]	(1)
Dessing et al., 2013, Netherlands [49]	*n* = 76, *n*_male_ = 32; *n*_female_ = 44 Age = 6 to 11 years; *M* = 8.6 (SD = 1.4) years	QStarz BT-Q1000X GPS, ActiGraph GT1M, 7 days	MVPA > 574 counts per 15 s epoch	Schoolyard (10 m buffer), Inside school (inside the school building)	Mean daily minutes of MVPA (SD) Schoolyard: Boys = 8.8 (5.1), Girls = 7.0 (5.1) Inside School: Boys = 4.9 (5.2), Girls = 7.1 (8.2)	(5)
Evenson et al., 2018, USA [50]	*n* = 265 girls, *n*_park visits_ = 73 Age = High school students (baseline 10th-11th grade)	Garmin Foretrex 201 GPS, ActiGraph model AM7164, 6 days	ST < 100 cpm LPA = 100–2999 cpm MVPA ≥ 3000 cpm MPA and VPA unclear	Parks (points within national, state and local parks and forests, excluding points within 50 m of participants residence)	Baseline mean, median (25th and 75th percentile) minutes of PA during park visits (*n* = 73) ST: mean = 22.3; median = 9.0 (25th percentile = 1.0, 75th percentile = 26.0) LPA: mean = 29.2; median = 12.0 (25th percentile =3.0, 75th percentile = 44.0) MPA: mean = 4.5; median = 1.0 (25th percentile = 0.0, 75th percentile = 3.0) VPA: mean = 0.9; median = 0.0 (25th percentile = 0.0, 75th percentile = 0.0) MVPA: mean = 5.4; median = 1.0 (25th percentile = 0.0, 75th percentile = 6.0)	(2)
Jones et al., 2009, UK [51]	*n* = 100; *n*_male_ = 47, *n*_female_ = 53 *n*_Urban_ = 68, *n*_Rural_ = 32 Age = 9 to 10 years	Garmin Forerunner 205 GPS, ActiGraph GT1M, 4 days	MPA = 2000–3999 cpm VPA ≥ 4000 cpm MVPA bouts ≥ 2000 cpm for 5 min	Neighborhood (area within 800 m along pedestrian network: roads plus designated public footpaths), buildings (included domestic residences, shops, indoor sports facilities, any other covered structures), areas of other built land (features such as car parks and yards, hard surface play areas, pedestrianized thoroughfares), roads and pavements, private gardens, parks, farmland, grassland, woodland, beaches	Mean minutes of MVPA per child across four days Inside Neighborhood: Total = 97.0, Boys = 115.6; Girls = 80.6; Urban 105.5; Rural = 79.1 Land use types Buildings: Total = 24.8; Boys = 29.0; Girls = 21.1; Urban = 28.3; Rural = 17.4 Other built land use: Total = 20.0; Boys = 22.3; Girls = 18.0; Urban = 22.4; Rural = 15.0 Roads and pavements: Total =16.1; Boys = 19.3; Girls = 13.4; Urban = 17.3; Rural = 13.8 Gardens: Total = 42; Boys = 54.2; Girls = 31.3; Urban = 46.9; Rural = 31.7 Parks: Total= 7.4; Boys= 10.4; Girls= 4.8; Urban = 6.5; Rural= 9.6 Farmland: Total = 14.5; Boys = 20.7; Girls = 9.0; Urban = 13.5; Rural = 16.6 Grassland: Total = 14.2; Boys = 16.2; Girls = 12.5; Urban = 3.6; Rural = 7.1 Woodland: Total = 4.4; Boys = 5.9; Girls = 3.0; Urban = 3.5; Rural = 6.1 Beaches: Total = 0.7; Boys = 0.7; Girls = 0.6; Urban = 0.4; Rural = 1.3 Mean minutes (SD) spent in MVPA bouts across four days Inside Neighborhood: Total = 24.9 (30.1); Boys = 34.9 (34.7); Girls = 16.0 (22.3); Urban = 25.7 (27.1); Rural = 23.1 (35.4) Land use types** Buildings: Total = 2.8 (6.0); Boys = 4.1 (6.9); Girls = 1.7 (8.1); Urban = 3.4 (7.0); Rural = 1.5 (2.9) Other built land use: Total = 5.5 (10.7); Boys = 6.5 (11.5); Girls = 4.6 (10.3); Urban = 6.5 (11.6); Rural = 3.5 (8.1) Roads and pavements: Total = 7.5 (11.7); Boys = 10.4 (14.5); Girls = 4.9 (8.2); Urban = 7.9 (11.0); Rural = 6.7 (12.9) Gardens: Total = 9.6 (16.5); Boys = 14.6 (21.0); Girls = 5.1 (9.5); Urban = 11.0 (15.5); Rural = 6.6 (17.6) Parks: Total = 2.9 (10.0); Boys = 3.8 (11.2); Girls = 2.1 (9.3); Urban = 2.3 (8.9); Rural = 4.2 (11.6) Farmland: Total = 5.4 (14.8); Boys = 9.3 (19.9); Girls = 2.0 (6.8); Urban = 3.8 (12.4); Rural = 8.8 (18.1) Grassland: Total = 4.7 (12.7); Boys = 7.5 (17.4); Girls = 2.3 (5.2); Urban = 3.6 (5.7); Rural = 7.1 (20.0) Woodland: Total = 1.2 (2.8); Boys = 1.5 (2.9); Girls = 0.9 (2.9); Urban = 1.1 (2.9); Rural = 1.4 (2.6) Beaches: Total = 0.2 (1.7); Boys = 0.1 (0.8); Girls = 0.3 (2.7); Urban = 0.1 (0.6); Rural = 0.5 (2.8) Significant difference between boys and girls at **p < 0.001	(1;2)
Klinker et al., 2014, Denmark [52]	*n* = 367, *n*_male_ = 175 (47.7%), *n*_female_=192 Age = 11 to 16 years; *M* = 13.2 (SD = 1.2) years	Qstarz BT-Q1000X GPS, ActiGraph GT3X, 7 days	MPA ≥ 2296 VPA ≥ 4012	School grounds (10 m buffer), Clubs (10 m buffer), Sport facilities (10 m buffer), Playgrounds (10 m buffer), Urban green space (10 m buffer), Shopping centers (10 m buffer), School, Home (10 m buffer)	Daily median minutes of MVPA (IQR) by gender School grounds (in leisure time): boys = 2.8 (1.5–7.3); girls = 2.2 (1.3–4.3)** Clubs: boys = 0.2 (0.0–1.3); girls = 0.0 (0.0–0.4) ** Sport facilities: boys =0.2 (0.0–4.8); girls = 0.0 (0.0–0.5) ** Playgrounds: boys = 0.0 (0.0–0.5); girls = 0.0 (0.0–0.3)* Urban green space: boys = 1.9 (0.5–4.4); girls = 1.5 (0.3–3.6)* Shopping center: boys = 0.0 (0.0–0.0); girls = 0.0 (0.0–0.0) School: boys = 24.9 (15.9–35.6); girls = 18.8 (13.0–25.8)** Home: boys = 4.8 (2.3–10.3); girls = 6.5 (3.0–12.8) Significant differences between boys and girls at *p < 0.05, and **p < 0.01 Daily median minutes of MVPA (IQR) by age School grounds (in leisure time): children = 3.3 (1.8–7.2); adolescents = 1.8 (1.0–3.1) Clubs: children = 0.0 (0.0–0.9); adolescents = 0.1 (0.0–0.5) Sport facilities: children = 0.0 (0.0–1.3); adolescents = 0.1 (0.0–1.3) Playgrounds: children = 0.0 (0.0–0.5); adolescents = 0.0 (0.0–0.3) Urban green space: children = 1.6 (0.3–3.6); adolescents = 1.8 (0.4–4.3) Shopping center: children = 0.0 (0.0–0.0); adolescents = 0.0 (0.0–0.0) School: children = 23.9 (17.1–34.9); adolescents = 17.5 (12.1–25.5) Home: children = 5.6 (2.6–13.3); adolescents= 5.5 (2.6–10.4)	(2;3;5)
Kneeshaw-Price et al., 2013, USA [53]	*n* = 682, *n*_male_ = 342, *n*_female_ = 340; Age = 6 to 11 years; *M* = 9.1 (SD= 1.6) years	Location logbook, ActiGraph GT1M, 7 days	MVPA ≥ 3MET (Freedson equation: MET = 2.757 + (0.00 15 × cpm) -(0.08957 × age (year)) -(0.000038 × cpm × age (year)))	Home (if parent lists “front yard” or “backyard” in place log; this is also considered home); School; Neighborhood (child active in the area around home or neighborhood but not at a specific place, no address needed); Others’ homes; Other schools; Public, outdoor parks and recreation facilities; Public, indoor recreation facilities, Private recreation facilities; Service locations, Shopping; Food eateries.	Average daily MVPA (SD) in minutes in each location Home: Total = 62.6 (36.7); Boys_6–8_ = 83.4 (40.4)^d^; Boys_9–11_ = 50.3 (31.2)^b^; Girls_6–8_ = 76.2 (33.8)^c^; Girls_9–11_ = 41.0 (21.3)^a^ School: Total = 37.6 (27.4); Boys_6–8_ = 46.9 (33.5)^d^; Boys_9–11_ = 34.4 (23.5); Girls_6–8_ = 41.4 (28.6); Girls_9–11_ = 28.0 (18.4)^a^ Others’ homes: Total = 10.1 (13.8); Boys_6–8_ = 12.6 (18.7); Boys_9–11_ = 9.2 (11.2); Girls_6–8_ = 11.1 (13.8); Girls_9–11_ = 7.7 (9.3) Service locations: Total = 8.0 (14.9); Boys_6–8_ = 12.0 (22.0)^d^; Boys_9–11_ = 6.2 (10.1); Girls_6–8_ = 9.0 (15.4); Girls_9–11_ = 4.9 (7.2)^a^ Public; outdoor parks; rec: Total = 6.9 (10.9); Boys_6–8_ = 8.5 (11.9)^d^; Boys_9–11_ = 8.9 (12.1)^d^; Girls_6–8_ = 5.8 (10.7); Girls_9–11_ = 4.2 (7.5)^a;c^ Shopping: Total= 3.2 (4.7); Boys_6–8_= 3.8 (5.7); Boys_9–11_= 2.1 (2.8)^b^; Girls_6–8_= 4.4 (5.9)^c^; Girls_9–11_ = 2.4 (3.0) Other Schools: Total = 3.3 (8.1); Boys_6–8_ = 3.4 (8.4); Boys_9–11_ = 4.7 (9.3); Girls_6–8_ = 3.1 (7.4); Girls_9–11_ = 3.1 (7.4) Food eateries: Total = 1.3 (2.3); Boys_6–8_ = 1.8 (3.0); Boys_9–11_ = 0.9 (2.0); Girls_6–8_ = 1.3 (2.2); Girls_9–11_ = 1.1 (1.9) Private rec. facilities: Total = 3.1 (6.4); Boys_6–8_ = 3.2 (6.5); Boys_9–11_ = 2.4 (4.8); Girls_6–8_ = 3.4 (6.0); Girls9–11 = 3.3 (7.9) Public; indoor rec facilities: Total = 1.8 (8.0); Boys_6–8_ = 3.0 (11.9); Boys_9–11_ = 1.7 (6.3); Girls_6–8_ = 1.8 (7.0); Girls_9–11_ = 0.9 (5.4) Neighborhood: Total = 2.1 (6.5); Boys_6–8_ = 2.0 (5.3); Boys_9–11_ = 2.6 (9.1); Girls_6–8_ = 1.7 (5.3); Girls_9–11_ = 2.1 (5.2) ^a^Significantly different from boys 6–8 years old; ^b^Significantly different from girls 6–8 years old; ^c^Significantly different from boys 9–11 years old; ^d^Significantly different from girls 9–11 years old *p* < 0.004	(1;2;3;5)
Lachowycz et al., 2012, UK [54]	*n* = 902, 52.5% female Age = 11 to 12 years	Garmin Fortrex 201 GPS on 4 school days (3pm-10pm) and 1 weekend day (8am-10pm), ActiGraph GT1M, 7 days	ST < 100 cpm LPA = 100–2296 cpm MVPA ≥ 2296 cpm	School grounds greenspace (land identified as grassland within area clearly defined as primary or secondary school); parks (formal: organized layout and structured path network aiming for aesthetic enjoyment, and generally well maintained; informal—design with emphasis on informal recreation; natural: habitats providing access to nature, such as heathland, woodland, wetland; young persons: areas designed for use by children or teenagers, including those with play and games equipment; sports: areas for organized and competitive sports, such as playing fields and tennis courts); private gardens; other greenspace (vegetated areas not defined as public parks, including private sports and recreation facilities, cemeteries, golf courses, gardens of publicly accessible buildings such as universities and hospitals); roads/pavements, green verges (small areas of vegetated land with grass or fragmentary vegetation, e.g., in center of roundabouts and narrow strips or banks of vegetation alongside pavements); built surfaces (car parks, pedestrianized thoroughfares)	Mean minutes of daily PA (SD) on weekdays (*n* = 614) Greenspace (overall): ST = 6.0 (16.1); LPA = 3.5 (7.9); MVPA = 2.4 (4.8) Parks (all types): ST = 1.1 (6.8); LPA = 1.2 (7.8); MVPA = 0.7 (4.7) Parks formal: ST = 0.2 (3.0); LPA = 0.3 (4.1); MVPA = 0.2 (3.3) Parks informal: ST = 0.5 (4.9); LPA = 0.4 (4.1); MVPA = 0.2 (1.6) Parks natural: ST = 0.1 (2.3); LPA = 0.1 (1.5); MVPA = 0.1 (1.1) Parks sports: ST = 0.1 (10.2); LPA = 0.1 (10.6); MVPA = 0.1 (7.4) Parks young persons: ST = 0.2 (4.0); LPA = 0.3 (6.6); MVPA = 0.1 (3.4) Private gardens: ST = 4.8 (15.1); LPA = 2.2 (4.2); MVPA = 1.6 (2.8) School grounds greenspace: ST = 0.1 (5.5); LPA = 0.1 (5.2); MVPA = 0.1 (3.3) Other greenspace: ST = 0.01 (0.5); LPA = 0.01 (0.5); MVPA = 0.01 (0.4) Roads/pavements: ST = 2.8 (7.2); LPA = 2.0 (3.7); MVPA = 1.9 (3.2) Green verges: ST = 0.3 (2.7); LPA = 0.2 (2.3); MVPA = 0.2 (1.8) Built surfaces: ST = 5.5 (12.4); LPA = 3.4 (6.1); MVPA =2.6 (4.4) Mean minutes of daily PA (SD) on weekend days (*n* = 301) Greenspace (overall): ST = 9.0 (26.9); LPA = 6.1 (15.7); MVPA = 3.5 (9.1) Parks (all types): ST = 3.4 (19.1); LPA = 3.5 (16.7); MVPA = 2.2 (10.5) Parks formal: ST = 0.5 (8.7); LPA = 0.7 (8.5); MVPA = 0.4 (4.3) Parks informal: ST = 1.0 (11.9); LPA = 1.1 (7.7); MVPA = 0.7 (5.0) Parks natural: ST = 0.7 (15.2); LPA = 0.6 (8.8); MVPA = 0.5 (6.6) Parks sports: ST = 0.1 (3.2); LPA = 0.1 (1.9); MVPA = 0.05 (1.2) Parks young persons: ST = 1.0 (19.1); LPA = 1.0 (13.9); MVPA = 0.6 (7.6) Private gardens: ST = 5.6 (23.4); LPA = 2.5 (7.7); MVPA = 1.2 (3.2) School grounds greenspace: ST = 0.1 (2.5); LPA = 0.1 (5.1); MVPA = 0.1 (1.8) Other greenspace: ST = 0.03 (1.3); LPA = 0.01 (0.4); MVPA = 0.01 (0.3) Roads/pavements: ST = 3.9 (12.5); LPA = 2.2 (7.6); MVPA = 1.6 (6.5) Green verges: ST = 0.6 (7.0); LPA = 0.5 (5.1); MVPA = 0.3 (2.7) Built surfaces: ST = 7.1 (14.1); LPA = 4.2 (9.3); MVPA = 2.2(7.1)	(1;2;5)
Matisziw et al., 2016, USA [55]	*n* = 134, *n*_male_ = 72, *n*_female_ = 62 Age = 9 to 12 years	Qstarz BT-1300 GPS, ActiGraph, 3 × 11 days	MVPA ≥ 2296 cpm	Park/open space vegetated, residential vegetated, commercial vegetated, industrial vegetated, agriculture vegetated, institutional vegetated, institutional built, residential built, commercial built, industrial built, park/open space built, transportation built, water	Average minutes of MVPA before school, after school, on weekends Park/open space vegetated: Before school = 0.4; After school = 17.6; Weekend = 17.5 Residential vegetated: Before school = 0.1; After school = 2.9; Weekend = 1.8 Commercial vegetated: Before school = 0.0; After school = 0.3; Weekend = 0.6 Industrial vegetated: Before school = 0.0; After school = 0.0; Weekend = 0.0 Agriculture vegetated: Before school = 0.0; After school = 0.7; Weekend = 0.5 Institutional vegetated: Before school = 0.0; After school = 0.0; Weekend = 0.0 Institutional built: Before school = 13.1; After school = 7.3; Weekend = 2.8 Residential built: Before school = 17.6; After school = 130.6; Weekend = 84.3 Commercial built: Before school = 0.6; After school = 11.1; Weekend = 9.6 Industrial built: Before school = 0.0; After school = 0.8; Weekend = 0.7 Park/open space built: Before school = 0.0; After school = 1.8; Weekend = 0.4 Transportation built: Before school = 0.1; After school = 0.4; Weekend = 0.3 Water: Before school = 0.0; After school = 0.1; Weekend = 0.1	(1;2)
Moore et al., 2014, UK [56]	*n* = 28, *n*_male_ = 11, *n*_female_ = 17 Age = 11 to 14 years; *M* = 11.8 years	QStarz BT-Q1000XT GPS, ActiGraph GT3X, 7 days	MVPA ≥ 2220 cpm (in bouts ≥ 3 minutes)	Home, school, street, rural/urban green space	Mean minutes of MVPA (SD) during 7-day period School: 40.2 (35.1) Streets: 28.1 (43.8) Home: 11.8 (18.2) Rural/Urban green: 4.8 (14.5)	(1;2;3;5)
Oreskovic et al., 2012, USA [57]	*n* = 24, 41.7% maleAge = 11 to 12years	Forerunner 201 GPS, ActiGraph GT1M, 3 × 7 days	MPA = 1952–5724 cpm VPA ≥ 5725 cpm	Home (25 m buffer), school (100 m buffer), park/playground, street/walking	Total minutes of MVPA Home: 670 School: 169 Park/playground: 217 Street/walking: 833	(1;2;3;5)
Oreskovic et al., 2015, USA [58]	*n* = 80, *n*_male_ = 35, *n*_female_ = 45 Age = 11 to 14 years; *M* = 12.6 (SD = 1.1) years	Qstarz BT-Q1000XT GPS, ActiGraph GT3X, 2 × 7 days	ST < 100 cpm MVPA ≥ 2296 cpm	Home (40 m buffer), school (40 m buffer), park, playground, street/sidewalk	Median daily minutes (IQR) of daily MVPA and ST (where reported) School: MVPA = 8 (5–12); ST = 87 (63–110) Home: MVPA = 4 (2–8); ST = 50 (40–69) Streets/Sidewalks: MVPA = 5 (3–9) Playground: MVPA = 3 (1–6) Park: MVPA = 2 (1–4)	(1;2;3;5)
Rainham et al., 2012, Canada [59]	*n* = 316, 47% female, *n*_Urban_ = 91, *n*_Suburban_ = 102, *n*_Rural_ = 123 Age = 12 to 16 years; *M* = 13.3 (SD = 0.92) years	EM-408 SiRF III 12-channel GPS, ActiGraph™ GT1M, 8 days	unclear	Home, School	Mean minutes of MVPA (SD) Home Urban: 20.8 (25.1); Low SES (*n* = 54) = 17.7 (19.9); High SES (*n* = 37) = 25.8 (30.8) Suburban: 20.0 (29.5); Low SES (*n* = 79) = 16.7 (25.2); High SES (*n* = 23) = 31.0 (39.5) Rural: 20.4 (29.2); Low SES (*n* = 73) = 22.0 (30.2); High SES (*n* = 50) = 18.0 (27.9) School Urban: 45.7 (45.2); Low SES (*n* = 54) = 39.2 (42.9); High SES (*n* = 37) = 55.3 (47.4) Suburban: 18.6 (28.0); Low SES (*n* = 79) = 16.0 (19.9); High SES (*n* = 23) = 27.6 (45.8) Rural: 29.8 (39.7); Low SES (*n* = 73) = 38.9 (46.8); High SES (*n* = 50) = 16.5 (20.1)	(3;5)
Remmers et al., 2019, NL [60]	*n* = 255 *n*_male_ = 117 *n*_female_ = 138 Age = 10 to 12; *M* = 12.1 (SD= 10.5) years	Qstarz BT-Q1000XT GPS, ActiGraph GT3X, 7 days afterschool time segment	LPA = 101–2295 cpm MVPA ≥ 2296 cpm	Home (within 10 m of self-reported residential parcel) School (within 10 m of geo-referenced parcel) Sports facilities (within 10 m of geo-referenced parcel) Shopping centers (within 10 m of geo-referenced parcel) Afterschool childcare (within 10 of geo-referenced parcel)	Unadjusted median daily minutes (IQR) of afterschool LPA Residential parcel (home): Total = 89.8 (57.4) School grounds: Total = 15.5 (33.0) Sports grounds: Total = 54.8 (34.7) Afterschool childcare: Total = 10.0 (21.7) Shopping centers: Total = 44.5 (58.5) Unadjusted median daily minutes (IQR) of afterschool MVPA: Residential parcel (home): Total = 8.3 (14.2);Boys mean= 2.8 (SD = 0.2); Girls mean = 3.7 (SD = 0.4)* School grounds: Total = 13.5 (35.0); Boys mean = 2.8 (SD = 4.9); Girls mean = 0.9 (SD = 0.1)* Sports grounds: Total = 41.0 (29.3); boys mean = 13.8 (1.3); girls mean = 7.7 (0.9)* Afterschool childcare: Total = 2.2 (13.2) Shopping centers: Total = 8.0 (11.0) Significant difference between boys and girls at * *p* < 0.05	(2;3;5)
Robinson et al., 2013, USA [61]	*n* = 31, *n*_male_ = 9, *n*_female_ = 22 Age = 11 to 14 years; *M* = 12 years	QStarz BT-Q1000XT GPS, ActiGraph GT3X, 2x7 days	MVPA ≥ 2296 cpm	Census-defined Neighborhood, Youth-identified neighborhood (defined for subjects as area(s) in which they live and where they spend their time)	Average daily minutes of MVPA Census-Defined Neighborhood: 9.5 Youth-Identified Neighborhood: 14.7	(1)
Van Kann et al., 2016, NL [62]	*n* = 257, *n*_male_ = 120, *n*_female_ = 137 Age = 8 to 11 years	QStarz, BT-Q1000XT GPS, ActiGraph GT3X, 5 days	ST < 101 cpm MVPA > 2295 cpm	Schoolyard (10 m buffer)	Mean daily minutes of MVPA (SD) on schoolyard Morning recess: Total (*n* = 172) = 1.97 (1.96); Boys (*n* = 82) = 2.43 (2.21); Girls = 1.55 (1.60)** Afternoon recess: Total (*n* = 167) = 4.83 (4.40); Boys (*n* = 76) = 5.88 (4.53); Girls (*n* = 91) = 3.96 (4.11)** Outside school hours: Total (*n* = 153) = 1.58 (1.88); Boys (*n* = 69) = 1.83 (2.18); Girls (*n* = 84) = 1.38 (1.58) Schoolyard total day: Total (*n* = 117) = 8.67 (6.34); Boys (*n* = 52) = 10.48 (6.59); Girls (*n* = 65) = 7.22 (5.79)** Mean daily minutes of ST (SD) on schoolyard Morning recess: Total = 4.23 (3.12); Boys = 3.51 (3.12); Girls = 4.89 (2.99)** Afternoon recess: Total = 8.95 (7.28); Boys = 8.67 (7.11); Girls = 9.18 (7.45) Outside school hours; Total = 6.38 (5.96); Boys = 6.54 (7.2); Girls = 6.26 (4.74) Schoolyard total day: Total = 20.16 (12.0); Boys = 18.82 (12.62); Girls = 21.24 (11.46) Significant differences between boys and girls at **p ≤ 0.01	(5)
Wheeler et al., 2010, UK [63]	*n* = 1053, *n*_male_ = 495, *n*_female_ = 558 Age = 10 to 11 years; *M* = 10.9 years	Garmin Fortrex 201 GPS, 4 of 7 days (school days between end of school and bedtime), ActiGraph GT1M, 7 days	MVPA ≥ 3200 cpm	Outdoors in greenspace area (within 400 km², of Bristol), outdoors not greenspace area (400 km²)	Mean daily minutes MVPA (SD) per personOutdoors; in greenspace: Boys = 2.48 (5.54); Girls = 1.47 (4.34) Outdoors; not greenspace: Boys = 5.52 (8.02); Girls = 4.72 (7.16) Total MVPA minutes across four days: Outdoors; in greenspace: Boys = 2218; Girls = 1477 Outdoors; not greenspace: Boys = 6306; Girls = 6281	(2)

Abbreviations: *n*, sample size; *n*, subsample; *M*, mean; GPS, global positioning system; ST, sedentary time; cpm, counts per minute; MVPA, moderate-to-vigorous physical activity; IQR, interquartile range; SD, standard deviation; MET, metabolic equivalent; SES, socio-economic status; bpm = beats per minutes; VPA, vigorous physical activity; LPA, light physical activity; MPA = moderate physical activity. Behavior settings: (1) Neighborhood environment; (2) Recreational Environment; (3) Home environment; (5) School environment. Note that only differences between sex/gender groups were marked as statistically significant, where applicable. Other group differences were not marked.

**Table 2:** Description of included studies targeting adults and summary of main findings.

Study Details	Participant Characteristics	Measurement Tools and Days of Data Collection	Physical Activity Cut-Points	Description of Locations	Minutes of Physical Activity in Identified Locations	Behavior Setting	
Baek et al., 2016, USA [64]	*n* = 129 women, *n*_Korean_ = 60, *n*_White_ = 69 Age = 20 to 60 years *M*_Korean_ = 39.1 (SD = 7.5) *M*_white_ = 47.5 (SD = 8.4)	GlobalSat DG-100 GPS, ActiGraph GT1M and GT3X, 7 days	PA bouts > 1000 cpm for at least 5 min	Home (< 50m buffer)	Mean minutes (SD) per day of home-based PA bout duration Korean immigrant women: 2.90 (4.50) White women: 11.50 (14.50)	(3)	
Evenson et al., 2013, USA [65]	*n* = 238, *n*_female_ = 134, *n*_male_ = 104 Age = 18 to 85 years *M* = 40.4 years; n_18-35_=114; n_36-59_=83, n_60-85_=41	Qstarz BT-Q1000X GPS, ActiGraph GT1M, 3 × 1 week	ST ≤ 100 cpm, LPA = 101–759 cpm lower MPA= 760-2019 cpm MPA = 2020–5998 cpm VPA ≥ 5999 cpm	Park (points falling within study parks excluding points within 50m of participant’s residence)	Mean and median (IQR) minutes of PA per day during park visits ST: mean = 9.9; median = 3.8 (0.6–11.0) LPA: mean = 5.1; median = 1.9 (0.3–5.5) Lower MPA: mean = 3.6; median = 1.0 (0.2–3.4) MPA: mean = 2.2; median = 0.5 (0.0–2.6) MVPA: mean = 2.3; median = 0.5 (0.0–2.7) VPA: mean = 0.1; median = 0 (0–0)	(2)	
Holliday et al., 2017, USA [66]	*n* = 223, *n*_male_ = 97, *n*_female_ =126 Age = 18 to 85 years	Qstarz BT-Q1000X GPS, ActiGraph GT1M, 3 × 7 days	PA bout minutes (cpm above cut-point ≥ 10 min): Matthew’s MVPA ≥ 760 cpm, NHANES MVPA ≥ 2020 cpm, NHANES VPA ≥ 5999 cpm	Participant homes, Roads, Parks, Commercial locations (stand-alone retail locations, strip malls, malls, dense commercial districts, restaurants, and gas stations), Schools (including pre-K to university), Fitness locations (pay gyms, private tennis/soccer facilities, swim clubs, dance/martial arts studios), Footpaths/trails, Residential locations (excluding the participant’s home)	Total and median/participant (IQR) PA bout minutes over 3 weeks Matthews’ MVPA Home: total = 42,375; median = 116 (40–242) Road: total = 21,885; median = 25 (0–105) Park: total = 19,465; median = 11 (0–72) Commercial: total = 12,375, median = 14 (0–42) School: total = 11,064; median = 0.0 (0–32) Fitness: total = 6092; median = 0.0 (0–0) Residential: total = 5053; median = 0.0 (0–17) Footpath/trail: total = 2016; median = 0.0 (0–1) NHANES MVPA and VPA Home: total MVPA = 9447; median = 6 (0–43) and total VPA = 994; median = 0 (0–0) Road: total MVPA = 12,820; median = 6 (0–48) and total VPA = 1250; median = 0 (0–0) Park: total MVPA = 5808; median = 0 (0–12) and total VPA = 227; median = 0 (0–0) Commercial: total MVPA = 1573; median = 0 (0–3) and total = 206; median = 0 (0–0) School: total MVPA = 4242; median = 0 (0–0) and total VPA = 634; median = 0 (0-0) Fitness: total MVPA = 3565; median = 0 (0–0) and total VPA = 1023; median = 0 (0–0) Residential: total MVPA= 1009; median = 0 (0-0) and total VPA= 112; median = 0 (0-0) Footpath/trail: total MVPA= 1352; median = 0 (0-0) and Total VPA= 478; median = 0 (0-0)	(1;2;3;5)	
Hurvitz et al., 2014, USA [67]	*n* = 611, *n*_male_ = 237, *n*_female_= 374 Age > 20 years, *n*_<40_ = 135, *n*_40-65_ = 395, *n*_>65_ =81	GlobalSat DG-100 GPS, ActiGraph GT1M, 7 days	ST ≤ 150 cpm LPA = 150–1951 MPA = 1952–5274 VPA ≥ 5275	Home (straight line distance < 125m), Near home (straight line distance < 125–1166m)	Mean daily minutes (SD) of ST Home: 183.3 (90.7) Near Home: 29.9 (25.1) Mean daily minutes (SD) of LPA Home: 68.6 (34.3) Near Home: 11.4 (9.4) Mean daily minutes (SD) of MPA Home: 7.1 (4.9) Near Home: 13.2 (9.0) Mean daily minutes (SD) of VPA Home: 2.0 (0.7) Near Home: 6.0 (2.5)	(1;3)	
Jansen et al., 2015, NL [68]	*n*_not-frail_ =74, *n*_male_ = 42, *n*_female_=32 Age = 65 to 89 years; *M* = 73.4 (SD = 6.1) years	Qstarz BT-Q1000X and BT-Q1000XT GPS, ActiGraph GT3X+, 7 days	ST = 0–50 cpm LPA = 51–759 cpm MPA = 760–1951 cpm VPA ≥ 1952 cpm	At home	Mean (SD) and median daily minutes (IQR) of PA ST: Mean = 370.32 (159.61); median = 380.58 (273.52–507.78) LPA: Mean = 54.19 (37.15); median = 49.36 (29.98–75.03) MVPA: Mean = 0.19 (0.27); median = 0.11 (0.02–0.27)	(3)	
Jansen et al., 2016, NL [69]	*n* = 308, *n*_female_ = 54.9% Age = 45 to 65; *M* = 56.4 (SD = 6.2) years	QStarz BT-Q1000XT GPS, ActiGraph GT3X+, 7 days	MPA = 3208–8564 cpm, VPA ≥ 8565 cpm, MVPA = MPA + VPA	Home (25m buffer), Other residential area (25m buffer around residence, land use > 70% residences), Residential and shopping area (25m buffer including residence, shops or foodservice industry), Shopping area (25m buffer, land use > 70% shops or foodservice industry), Workplace (50m buffer from work address or 25m buffer from health care institutions, offices, educational institutions, lodging, industry or shops according to building data if participants spent ≥ 240 min at location), Small green area (parks and public garden or allotment garden), Larger green area (recreational or agricultural area, forest or natural terrain), Sports facilities (require membership/subscription; 10m buffer around sport facility)	Median (IQR) minutes of daily MVPA Home: 10.4 (16.8) Other residential area: 5.0 (14.2) Residential and shopping area: 0.6 (2.8) Shopping area: 1.0 (4.1) Small green area: 1.0 (10.1) Larger green area: 0.9 (6.6) Sports facilities: 4.2 (19.6) Workplaces: 9.9 (19.6)	(1;2;3;4)	
Miralles-Guasch et al., 2019, Spain [70]	*n* = 63, *n*_male_ = 55.6% Age = 65 to >75 years; *M* = 81.1 years	Qstarz Q-1000XT GPS, ActiGraph GT3X, 7 days	ST < 216 vector magnitude cpm Active > 216 vector magnitude cpm	Urban green spaces and areas within urban green spaces depending on vegetation type (i.e., forest, shrubland, grassland and surface type (pavement, gravel, mix surfaces)	Median minutes (total) in urban green spaces Male: ST = 6.5; active time = 3.5 Female: ST = 6.0; active time =3.5 Age 65–75 years-old: ST = 5.8; active time = 3.9 Age > 75 years-old: ST = 7.0; active time = 2.0 Distance urban green space from home: < 300m: ST = 6.1; active time = 1.9 301–600m: ST = 3.0; active time = 7.5 > 601m: ST = 4.0; active time = 5.3 <50,000m^2^: ST = 6.3; active time = 3.1 Median minutes (total) in different areas within urban green spaces Forest: ST = 2.8; active time = 2.0 Shrubland: ST = 4.5; active time = 1.7 Grassland: ST = 1.8; active time = 0.5 Pavement: ST = 2.1; active time = 1.5 Mix surfaces: ST = 3.3; active time = 2.3 Gravel: ST = 3.2; active time = 1.4	(2)	
Ramulu et al., 2012, USA [71]	*n* = 35, *n*_female_ = 74% Age = 18 to 61; *M* = 38	Brickhouse securities pTrac Pro GPS, Actical Accelerometer, 6 days	MVPA ≥ 1535 cpm	Home region (536m buffer)	Median minutes (IQR) of MVPA Weekday home region: 0 min (0–2) Weekend days home region: 1 min (0–4)	(3)	
Stewart et al., 2018, USA [72]	*n* = 634, *n*_male_ = 234 Age = 18 to > 65 years *n*_<45_ = 223, *n*_45-64_ = 318, *n*_≥65_ = 93	GlobalSat DG-100 GPS, ActiGraph GT1M, 7 days	PA bouts > 500 per 30 s for at least 5 min	Home neighborhood park (833m street network buffer), non-home neighborhood park (visits to parks completely outside the home neighborhood buffer)	Mean (SD) daily minutes of PA bouts Home neighborhood park PA: 1.3 (4.7) Non-home neighborhood park PA: 3.4 (7.7)	(2)	
Triguero-Mas et al., 2017, Spain, UK, NL, Lithuania [73]	*n* = 408 (*n*_female_ = 53.68%); *n*_Spain_ = 107 (*n*_female_ = 46.73%); *n*_UK_ = 92 (*n*_female_ = 56.52%); n_Netherlands_ = 105 (*n*_female_ = 57.14%); *n*_Lithuania_ = 104 (*n*_female_ = 54.81%) Age = 18 to 75 years; median (IQR) = 51.00 (26.00)	smartphone with integrated GPS receiver and motion sensor, CalFit application, 7 days	MVPA ≥ 3 MET (Freedson equation: MET= 1.439008 +(0.000795 x cnts·min-1)	PA in natural outdoor environment = green or blue space within 50 m of each location point	Median (IQR) daily minutes of MVPA Weekdays (*n* = 350) Natural outdoor environment: Total: 7.73 (19.25); Spain: 4.20 (9.40); UK: 4.60 (12.31); Netherlands: 21.00 (33.80); Lithuania: 8.57 (17.70) Weekends (*n* = 308) Natural outdoor environment: Total: 7.75 (24.12); Spain: 6.00 (15.88); UK: 4.00 (10.50); Netherlands: 25.50 (31.75); Lithuania: 6.00 (19.25)	(2)	

Abbreviations: *n*, total sample; *n*, subsample; *M*, mean; GPS, global positioning system; PA, physical activity; cpm, counts per minute; SD, standard deviation; ST, sedentary time; LPA, light PA; MPA, moderate PA; IQR, interquartile range; MVPA, moderate-to-vigorous PA. Behavior settings: (1) Neighborhood environment; (2) Recreational Environment; (3) Home environment; (4) Workplace environment; (5) School environment. Note that only differences between sex/gender groups were marked as statistically significant, where applicable. Other group differences were not marked.

## Data Availability

Data sharing not applicable.

## Supplementary Materials

The following are available online at https://www.mdpi.com/1660-4601/18/3/1240/s1, Table S1: PRISMA 2009 Checklist.
